# A Comparison of Willingness to Pay to Prevent Child Maltreatment Deaths in Ecuador and the United States

**DOI:** 10.3390/ijerph10041342

**Published:** 2013-03-28

**Authors:** Phaedra S. Corso, Justin B. Ingels, M. Isabel Roldos

**Affiliations:** 1 College of Public Health, University of Georgia, 110 E. Clayton Street, Suite 300, Athens, GA 30602, USA; E-mail: jingels@uga.edu; 2 School of Medicine, Universidad San Francisco de Quito, Quito EC170157, Ecuador; E-Mail: mroldos@usfq.edu.ec

**Keywords:** child maltreatment (CM), economic evaluation, willingness-to-pay (WTP), benefit-cost analysis (BCA), contingent valuation (CV) surveys

## Abstract

Estimating the benefits of preventing child maltreatment (CM) is essential for policy makers to determine whether there are significant returns on investment from interventions to prevent CM. The aim of this study was to estimate the benefits of preventing CM deaths in an Ecuadorian population, and to compare the results to a similar study in a US population. The study used the contingent valuation method to elicit respondents’ willingness to pay (WTP) for a 1 in 100,000 reduction in the risk of CM mortality. After adjusting for differences in purchasing power, the WTP to prevent the CM mortality risk reduction in the Ecuador population was $237 and the WTP for the same risk reduction in the US population was $175. In the pooled analysis, WTP for a reduction in CM mortality was significantly impacted by country (*p* = 0.03), history of CM (*p* = 0.007), payment mechanism (*p* < 0.001), confidence in response (*p* = 0.014), and appropriateness of the payment mechanism (*p* < 0.001). These findings suggest that estimating benefits from one culture may not be transferable to another, and that low- and middle-income countries, such as Ecuador, may be better served by developing their own benefits estimates for use in future benefit-cost analyses of interventions designed to prevent CM.

## 1. Introduction

Economic evaluation methods, such as benefit-cost analysis (BCA), are increasingly being used to show returns on investment of violence prevention interventions. BCA includes a comparison of an intervention’s costs to an intervention’s benefits measured in monetary units. The challenge, however, in using BCA is trying to put a monetary value on the benefits of these interventions, such as preventing child maltreatment (CM) morbidity or mortality [1,2]. While the field has made considerable progress in estimating the value of preventing CM events from a human capital perspective (e.g., valuing medical costs and productivity losses averted) [2,3,4,5,6], these estimates are invariably an underestimate of the true benefit of preventing a case of CM because long-term effects are hard to quantify, impacts on quality of life are not included, and it fails to capture any intangible benefits that may be at least as important to taxpayers [6,7].

Another methodology to value the benefits of preventing violent outcomes for use in a BCA is the contingent valuation (CV) method. The CV methodology relies on surveys to elicit the subjective value that individuals place on public goods that are not traded in the marketplace, such as clean air or clean water, or for a violence-free society in our example [8]. The logic of the CV method is that respondents are asked to think about the public good as if it could be purchased in the marketplace (that is, contingent upon an actual market), and then they are asked how much they would be willing to pay for it. As such, respondents’ willingness-to-pay (WTP) amount is a reflection of the value they place on the public good, that can thus be used as the benefits measure in a BCA. For example, Ludwig and Cook [9,10], in a CV study of respondents being asked to value the prevention of violence caused by firearms, found that respondents’ total WTP to reduce gun violence by 30% equaled $1.2 million per injury (in 1998 US$). Coupled with the programmatic costs associated with preventing gun violence, this benefits measure could be very useful in a future BCA of a gun violence prevention intervention.

Other studies have also applied the CV methodology to estimate the benefits of preventing violent outcomes [11,12,13]. For example, Cohen, Rust, Steen and Tidd [12] asked respondents if they would be willing to vote for a proposal requiring each household in their community to pay a certain amount to be used to prevent one in ten crimes in their community. Atkinson, Healey and Mourato [11] similarly asked respondents how much they would be willing to pay to reduce their risks for various types of crimes and their risks of victimization.

Only a few studies have used the CV approach for valuing the benefits of preventing CM. Corso *et al.* [14] valued the benefits of preventing CM deaths and Shadoin [15] valued how much of $100 respondents would be willing to pay for programs to prevent child abuse deaths, sexual abuse and physical abuse. The former study reported a mean WTP per person of $148 (in 2008 US$) and a median of $151 (in 2008 US$), for a 1 in 100,000 reduction in the mortality risk associated with CM. The latter study reported a significant positive correlation between WTP for prevention of CM and both knowledge of child CM and self-reported empathy of the respondent.

The application of the CV methodology as a benefits measure in BCAs is quite limited outside the United States, and virtually non-existent in the field of violence prevention. Perhaps because of this realization, the World Bank [1] and others [16] note the value of being able to transfer benefits obtained from the United States to low- and middle-income countries using the CV method by stating that “the simplicity of CV methodology allows for application of the same questionnaire, implying the same set of changes in outcomes, to be applied in different areas, regions, or countries [1].” In contrast, valuing the benefits of violence prevention based on medical costs averted is not as readily transferable across countries because of differences in national health care systems, public health interventions, and criminal justice systems. 

To that end, in this study we apply, for the first time ever, the CV methodology to assess the benefits of preventing CM mortality in the middle-income country of Ecuador. Second, we compare the values that respondents in Ecuador place on preventing CM deaths with values previously obtained in the United States [14], to determine if it is possible to transfer the benefits obtained from one country to another for use in BCAs. 

## 2. Methods and Results

### 2.1. Data Collection

The initial CV survey to elicit WTP to prevent CM deaths in the United States was conducted by telephone interview in Fall 2008 among a randomly selected sample of adult residents. A full description of the study sample, methods, and results of the US study are published elsewhere [14]. For this study, the original CV survey conducted in English in the United States was translated into Spanish and conducted by in-person interviews between February and June 2012, with a convenience sample of adult residents living in two cities in Ecuador, Quito and Guayaquil. Participants were recruited at utility payment centers in both cities to capture different social demographic gradients. Utility payment centers are located across the cities in different localities including shopping malls, community centers, and small shops. Following consent, participants were taken to a secluded area to conduct the survey. The questions were read to each participant and the research team recorded their answers on a paper version of the survey. Less than 1% of potential participants declined the survey. Each survey took between 15 and 20 min, and as compensation participants were given a $15 phone card.

The CV survey included several separate verbal protocols to establish the “contingent” market. For example, respondents were first presented with a range of questions asking their opinion about maternal and child health issues, before being asked to state their WTP to reduce CM deaths in their community. Respondents were given the following prompt:

*“Now imagine* *there is a program available to your city or town that is proven to be effective in reducing the risk of a child being killed by a parent or caretaker by 50%. This means that the number of children killed on average by child maltreatment in your community is reduced from 2 per 100,000 to 1 per 100,000 per year.”*

If the respondent was randomized to a question about a payment mechanism through taxes, the following question was asked.

*“If* *this program were available to your city or town, would you be willing to pay [randomly selected dollar amount] in extra taxes per year to sponsor this program given your household income and other expenses?”*

Otherwise, the respondent was asked about a payment mechanism through donations and the following question was asked.

*“The prevention program will* *be paid for by contributions from area residents, and cannot be started unless a minimum amount of money is raised from people like yourself. Would you be willing to donate [randomly selected dollar amount] to sponsor this program given your household income and other expenses?”*

We used the double-bounded dichotomous choice CV method [8]. With this approach, we first provided respondents with an initial WTP value, to which they responded “yes” or “no”, followed by a second WTP value that was either higher (yes) or lower (no) than the first WTP value, depending on the answer to the first question. To correct for potential starting point bias [8], the initial WTP values of $10 to $300 (in Ecuador US$) were randomly selected, and pre-determined in a pilot study. The second WTP values presented were either $25 higher (if first response was “yes”) or $25 lower (if first response was “no”). By comparison, in the US study [14], the initial WTP values were $25 to $275 (in US$) and the second bid value was randomly selected as some amount higher (or lower) than the initial bid value, not constrained by $25 more or less.

Because the literature [9,10] suggests that WTP for a risk reduction in mortality caused by violence is influenced by exposure to violence and the perception of the violence in one’s own community, the survey also asked respondents to describe their perception of CM prevalence in their community, and to report their exposure to abuse and neglect during childhood. Perception of CM risk in the neighborhood was coded as a categorical variable and equal to 1 if respondents reported that the risk was “somewhat less” or “much less” than average, equal to 2 if the neighborhood risk was considered “about average”, and equal to 3 if the risk was considered either “much greater” or “somewhat greater” than average. History of CM was coded as a dichotomous variable and equal to 1 if the respondent reported any act of physical abuse, sexual abuse, or neglect during childhood, and equal to 0 otherwise. 

To test for bias in the amount respondents’ were willing to pay for reductions in CM deaths based on the mechanism of payment, we asked respondents in a split sample either WTP in taxes or WTP in donations. We also asked about the appropriateness of the payment vehicle and how confident they were in their responses. In the comparison CV study conducted by Corso, Fang and Mercy [14], the authors found that payment mechanism did make a difference in WTP, with taxes producing significantly higher WTP compared to donations. These results are corroborated by Wiser [17] who found that respondents typically stated a higher WTP when confronted with a collective payment mechanism compared to a voluntary payment mechanism.

Finally, we collected data on several socio-demographic characteristics that have been shown to influence WTP in other studies, including age, gender, race/ethnicity, and income. For example, there is strong evidence that income, which is correlated with age, gender, and race/ethnicity, significantly and positively affects WTP [18]. We added both age and age squared into the regression to account for the possible non-linear relationship between age and WTP [19,20,21].

### 2.2. Analysis

We did not directly observe a respondent’s WTP for a reduction in mortality related to CM. However, we assumed a WTP value Y_i _* that is represented by the model Y_i _* = *X_i_*β + *ε_i_*, where the *ε_i_* are normally distributed with a mean zero and the *X_i_* represent individual respondent characteristics. While *Y_i_* * was not directly observed for respondent *i*, it is known to lie in the interval [*Y_i1_*, *Y_i2_*] based on responses elicited in the CV survey and the corresponding likelihood contribution is:

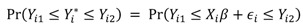
(1) 
When an upper bound is unknown (right-censored data) the likelihood contribution is:

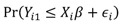
(2)
and when a lower bound is unknown (left-censored data), we set a lower bound of zero, and the likelihood contribution is:

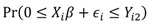
(3)


The maximum likelihood function was estimated with interval regression using the *intreg* command in Stata version 12 (Stata Corp, College Station, TX, USA).

The 2012 US$ reported in the Ecuador study were modified to reflect the purchasing power parity (PPP) of Ecuador$ compared to the PPP of US$, 0.55 [22]. The PPP is the amount of Ecuador US$ needed to purchase a similar amount of goods and services that $1.00 would buy in the United States. Therefore, a lower bound of $100 in Ecuador US$ is divided by 0.55 for a result of $181.82 in US$.

Second, to test for cross-country differences in WTP to prevent CM deaths, we combined the Corso, Fang and Mercy [14] CV survey data from the United States with the survey data collected in Ecuador for this study. To make values elicited from the two populations comparable, the 2008 US$ from the US respondents were converted to 2012 US$ using the all-items component of the Consumer Price Index [23]. We used interval regression with the combined datasets to test for a significant difference in the WTP values reported by the two groups. Demographic and covariate values were included in the combined analysis to control for differences in the two study groups. Finally, we estimated the mean WTP value for each country’s sample using interval regression without any demographic or covariate variables (referred to herein as “covariate unadjusted”). Bootstrapped standard errors (1,000 replications) were used to calculate bias-corrected 95% confidence intervals (CIs) on the mean WTP [24]. All results presented herein are in 2012 US$.

### 2.3. Results

Table 1 provides a summary of the demographic characteristics and other covariates of the Ecuador respondents for the N = 290 respondents that completed the CV survey on WTP to prevent CM deaths within the community. Most of the respondents were of Mestizo ethnicity (84.8%) and younger than 35 years of age (mean = 32.6). The respondents were evenly split by gender (50.3%) and CM history (50.7%). Household income was reported by the respondents as follows: 51.7% reported an income of $5,000 or less, 26.9% reported an income greater than $5,000 but less than $10,000, 10.7% reported an income greater than $10,000 but less than $15,000, and 10.3% reported an income greater than $15,000. Respondents reporting a household income of less than $15,000 were classified as low income (89.3%), and the remaining were classified as high income (10.3%), while only one respondent did not report income. Approximately 65.9% of the respondents believed the risk of CM in their community was greater than or equal to the average risk presented (2 per 100,000); 12.1% of the respondents perceived a lower risk. A little more than half (56.9%) of the study population felt confident in their ability to pay for a CM program at the values presented; 60.7% of the respondents stated that the payment mechanism with which they were presented was the appropriate mechanism for funding such a program. The population of Ecuador is 71.9% mestizo while the mean adult age is around 40 years of age and 51% female [25]. The CV survey respondents were younger than the general adult population but with a similar ethnic and gender breakdown.

**Table 1:** Respondent Demographics: Ecuador (N = 290), United States (N = 199) and Combined, Ecuador and United States (N = 489).

		Ecuador Respondents ^a^	US Respondents ^a^	Diff. *p*-value ^b^	Ecuador + US ^a^
*Age, years*		32.6 (10.9)	48.9 (17.2)	<0.001	39.3 (16.0)
*Female*		50.3	68.8	<0.001	57.9
*Race/Ethnicity*				<0.001	
	Mestizo	84.8	0.0		50.3
	White	9.3	64.3		31.7
	Black	4.5	25.6		13.1
	Other	1.3	11.1		5.3
*Household income, $ ^c^*				<0.001	
	Low	89.3	12.1		51.5
	High	10.3	65.8		39.3
	Missing	0.03	22.1		9.2
*CM history*		50.7	27.6	<0.001	41.3
*Neighborhood CM risk*				<0.001	
	<Average	12.1	45.7		25.8
	About average	21.1	31.7		26.0
	>Average	65.9	17.1		46.0
	Missing	0.0	5.5		2.2
*WTP by increased taxes*		44.8	53.8	0.052	48.5
*Confident in ability to pay*		56.9	55.8	0.807	56.4
*Appropriate payment mechanism*		60.7	70.4	0.028	64.6

^a ^Mean (standard deviation) reported for age and percentage of respondents for all other variables. ^b ^Based on two-sample *t*-tests for age and chi-square tests for the other variables. ^c ^Income levels used in comparing respondents from Ecuador and the United States. The Ecuador respondents reporting non-PPP adjusted income of $15,000 (PPP-adjusted ≈ $27,000) or less classified as low and all other non-missing as high. For the United States, respondents reporting income of $25,000 or less were classified as low and all other non-missing as high.

The demographics and other covariates of US respondents (N = 199) and for respondents in the combined (Ecuador and US) analysis are also reported in Table 1 (N = 489). The Ecuador and US respondents differed in a number of ways, based on a two-sample *t*-test for age, and chi-square tests for the other variables. The US respondents were significantly different from the Ecuador respondents in most demographic categories. They were, on average, 16 years older (*p* < 0.001), wealthier (*p* < 0.001), and included mostly white (*p* < 0.001) and female respondents (*p* < 0.001). The cutoff for high and low income of $25,000 was selected to make the reported incomes comparable. For Ecuador, an income of $15,000 when PPP-adjusted is near $27,000. Therefore, defining respondents from Ecuador with a household income below $15,000 is roughly comparable to respondents in the United States with a household income below $25,000. 

The US respondents were also much less likely to report any history of abuse (*p* < 0.001) compared to the Ecuadorian respondents. Further, a higher proportion of the US respondents believed that the CM risk in their own neighborhood was less than average (*p* < 0.001), and a higher proportion believed the payment mechanism they were provided was appropriate (*p* = 0.028). However, both groups reported similar confidence in their ability to pay the presented value (*p* = 0.807).

Table 2 presents the results of the interval regression using data from the CV survey conducted in Ecuador. The likelihood ratio chi-square (30.95, *p* = 0.003) indicates that the model is statistically significant compared to the constant-only model.

**Table 2:** Interval Regression Results of WTP for Reducing the Risk of CM Mortality by 1 in 100,000: Ecuador (N = 290).

	Regression Coefficient	*p*-value
Age	3.42	0.515
Age squared	−0.05	0.472
Non-Mestizo (Ref)	1.00	
Mestizo	12.08	0.642
Male (Ref)	1.00	
Female	11.13	0.568
Neighborhood CM risk < average (Ref)	1.00	
Neighborhood CM risk > average	26.19	0.388
Neighborhood CM risk about average	−0.09	0.998
Low household income (Ref)	1.00	
High household income	17.86	0.490
No history of CM (Ref)	1.00	
History of CM	−55.96	0.018
Pay through donation (Ref)	1.00	
Pay through taxes	44.85	0.017
Not confident in ability to pay for program (Ref)	1.00	
Confidence in ability to pay for program	−63.75	0.001
Not appropriate payment mechanism (Ref)	1.00	
Appropriate payment mechanism	36.17	0.064
Constant	172.41	
Likelihood ratio chi-square (df)	30.95 (13)	0.003

The constant term from the regression, reported in Table 2, corresponds to the estimated WTP value if a respondent was age 0 and reported the reference level for the other demographic covariates. Therefore, the regression coefficients reported in Table 2, estimate the adjustment to the reference WTP value for a respondent reporting demographics other than the reference level. To validate these estimates of WTP, economic theory [8] predicts that the proportion of “yes” responses to the initial bid value should decrease with an increasing bid value. For the Ecuador respondents, 100% with an initial bid of $10 responded “yes” which decreased almost monotonically to a “yes” response rate of 11% with an initial bid of $300. The results of the interval regression using data from the US population are reported elsewhere [14].

There were three significant predictors (*p* < 0.05) of WTP to reduce CM deaths in the Ecuador population: history of CM (*p* = 0.018), payment through taxes (*p* = 0.017), and confidence in ability to pay (*p* = 0.001). Respondents reporting any history of CM were willing to pay $56 (or 24%) less than those without a history of abuse. A similar (20% less), but non-significant, result was found with US respondents [13]. Respondents asked to pay taxes were also willing to pay $36 (or 21%) more than those asked to make charitable donations. A much larger significant result was found with US respondents [13], where respondents asked to pay taxes were willing to pay twice as much as those asked to pay in donations. Respondents who were confident in their ability to pay the presented value were willing to pay roughly $64 (or 27%) less than respondents lacking confidence. This was not a significant predictor reported for the US respondents [14].

In the interval regression for the combined analysis, the likelihood ratio chi-square (71.73, *p* < 0.001) indicates that the model is statistically significant compared to the constant-only model. The coefficient specifying country was significant (*p* = 0.031) and equal to −63.87 (see Table 3). This result suggests that the US respondents were willing to pay almost $64 (or 27%) less than the Ecuador respondents, for the same reduction in the risk of death associated with CM, after adjusting for demographic and other covariates. Four significant predictors (*p* < 0.05) of WTP were found for the combined analysis, as reported in the interval regression results in Table 3: history of CM (*p* = 0.007), payment through taxes (*p* < 0.001), confidence in ability to pay (*p* = 0.014), and appropriateness of the payment mechanism (*p* < 0.001).

**Table 3:** Interval Regression Results of WTP for Reducing the Risk of CM Mortality by 1 in 100,000: Ecuador and the United States (N = 498).

	Regression Coefficient	*p*-Value
Ecuador (Ref)	1.00	
**US**	**−63.87**	**0.031**
Age	−1.53	0.524
Age squared	0.02	0.557
Non-White/Non-Mestizo (Ref)	1.00	
White	16.66	0.418
Mestizo	27.93	0.304
Male (Ref)	1.00	
Female	−2.82	0.842
Neighborhood CM risk < average (Ref)	1.00	
Neighborhood CM risk > average	29.08	0.129
Neighborhood CM risk about average	−1.20	0.949
Low household income (Ref)	1.00	
High household income	16.25	0.417
No history of CM (Ref)	1.00	
History of CM	−43.37	0.007
Pay through donation (Ref)	1.00	
Pay through taxes	54.52	<0.001
Not confident in ability to pay for program (Ref)	1.00	
Confidence in ability to pay for program	−33.74	0.014
Not appropriate payment mechanism (Ref)	1.00	
Appropriate payment mechanism	63.34	<0.001
Constant	203.12	
Likelihood ratio chi-square (df)	71.73 (16)	<0.001

Similar interval regression analyses were carried out for respondents asked to consider the donation and taxes payment mechanisms separately (results not shown). The coefficient specifying country for the donation group was significant (−72.4; *p* = 0.050), yet was not significant for the taxes group (−61.4; *p* = 0.193). Therefore, we considered including in the full regression an interaction term of payment mechanism by country. This interaction term was not significant, but due to the small sample size and the limited power to detect differences in this population, we cannot make any definitive conclusions from this result.

Finally, Table 4 presents the covariate unadjusted mean estimates of WTP for a 50% reduction in the risk of death associated with CM for the Ecuador and US respondents. For the Ecuador respondents, the mean estimated WTP was $237 (95% CI = $215, $259). For the US respondents, the mean estimated WTP was $175 (95% CI = $154, $199). The difference in mean WTP between Ecuador and US respondents was $62, which is similar to the estimated difference from the pooled regression analysis reported in Table 3. Furthermore, the 95% CIs around the mean WTP values from the combined analysis do not overlap, further evidence of a significant difference in the WTP estimates.

**Table 4:** Estimated Mean WTP and 95% CI for Reducing the Risk of CM Mortality by 1 in 100,000: Ecuador and the United States.

	Mean WTP ^a^	95% CI ^b^
Ecuador (N = 290)	$237	$215, $259
US ^c^ (N = 199)	$175	$154, $199

^a ^All WTP values are expressed in 2012 US$, based on a PPP of 0.55 Ecuador$ per US$. ^b ^Bias-corrected CIs based on 1000 bootstrap replications. ^c ^Mean estimate differs from that reported by [14] as that estimate was covariate-adjusted, in 2008 US$, and the lower bound treated as missing rather than a zero (see Equation 3).

## 3. Discussion

This article estimates the benefits measure for reductions in CM deaths using CV methods in an Ecuadorian population, as compared to previous estimates obtained from a US population. The findings suggest that WTP values are different between the two countries, even when making the necessary PPP adjustments, with the Ecuador population valuing a 1 in 100,000 CM mortality risk reduction of $237 and the US population valuing the same risk reduction as $175. Our results are similar to those in Alberini *et al.* [26,27,28], Carlsson *et al.* [29], Ready *et al.* [30], and Rozan [31] who showed that WTP for the same mortality risk reductions differed across countries in ways not related to observable characteristics. For example, Alberini, Hunt and Markandya [27] found that WTP to reduce mortality risks remained significantly different across populations in the United Kingdom, Italy and France, even after WTP bids and household income figures were converted into PPP-adjusted amounts.

In fact, Alberini *et al.* [32] argue that preferences and WTP may differ across countries for cultural, income and access issues:

*“There is no reason why the preferences* *of people in other countries should be identical to preferences in the United States. Cultural factors, especially those that affect perceptions of illness, may alter people’s willingness to trade income for health. Along with income differentials, differences in health and educational status and the availability and cost of health care are some additional reasons to expect differences in such trade-offs.”*

This suggests that cultural differences and different sensitivities to the “good” being valued may result in systematic differences in WTP, even after controlling for observable characteristics of the populations.

One hint of a cultural difference between the United States and Ecuador in this assessment of CM mortality is the significant difference in the proportion of respondents indicating abuse or neglect as a child, with nearly 50% of the Ecuadorian population reporting exposure to CM and a little more than 25% of the US population reporting exposure. This self-reported history of child abuse could be a real difference in population-level prevalence of CM that might impact WTP, a difference in the comfort level of reporting abuse, or cultural differences in how CM is defined. Without having actual records of CM available for these respondents or follow-up questions to ask about comfort level with the survey questions, the true prevalence of CM in this population is not known. However, assuming that the reported CM rates are an accurate reflection of the prevalence of CM in the study population, one might expect that WTP to prevent CM would be higher based on the greater knowledge and exposure to the risk under assessment. For example, Leiter and Pruckner [33] and Deshazo and Cameron [34] have reported that exposure to a risk significantly and positively impacts WTP to reduce that risk. In contrast, we report that exposure to CM significantly decreased WTP to prevent CM deaths in the Ecuadorian population. In the other two studies of WTP to prevent CM mortality in the United States [14,15], one found that personal history of abuse decreased WTP to prevent CM deaths [14] and the other [15] found that history of abuse increased WTP to prevent CM deaths, yet neither were significant.

Alternatively, it could be that the Ecuadorian population is willing to pay more to prevent CM deaths because they believe that the risk is much greater than what is being reported in the “contingent” market described in the survey. For example, Hunter *et al.* [35] found that for respondents presented with a CV survey who perceived the presented risks as being lower (higher) than the actual risks, they consequently reported significantly lower (higher) WTP values. In this study, 66% of the Ecuadorian respondents stated that they perceived the CM mortality risk to be greater than the average risk (2 in 100,000) reported in the survey, whereas only 17% of the US respondents reported that they perceived the risk to be greater than the reported average. Therefore, following Hunter *et al.* [35], we might expect for the WTP to prevent CM mortality to be higher in the Ecuadorian population compared to the US population because of the perception of underlying risk in the community. However, as shown in Table 2, perception of risk did not significantly impact WTP to prevent CM deaths in the full regression of the Ecuador data. The same result holds true for the US respondents [14].

Where the WTP estimates from Ecuador and the United States coincide is the impact of payment mechanism on WTP to prevent CM. In both populations, those respondents asked to pay in taxes (*versus* donations) were willing to pay more to prevent CM deaths. As corroborated by Wiser [17] and others [36,37], taxes as a payment vehicle are more acceptable when considering goods that affect the whole of society, like violence.

Two important caveats to our study are that the Ecuador sample is not a national, randomly selected sample and the method of data collection differed between the two comparison studies. First, although the comparison sample from the United States [14] was also from a single geographic region, respondents were randomly selected. Thus, these results should be generalized to the broader national populations respectively, with caution. Second, the interview format, which was face-to-face in Ecuador compared to via telephone in the United States, may bias the WTP estimates if respondents are influenced by what they think the interviewer wants to hear, or if respondents experience embarrassment or shame in self-reporting abuse. In either case, it is not clear in which direction the WTP to prevent CM deaths would be biased. Although there are other limitations with the CV method for estimating WTP to prevent mortality risks in general (like stated preferences being higher than what persons might actually pay out-of-pocket), these biases would be present in both studies because the same CV instrument was used, and therefore not relevant to the cross-country comparison conducted here.

## 4. Conclusions

An important question when performing BCAs of CM interventions in low- to middle-income countries is whether estimates of WTP computed in the United States can be used as a benefits measure. Administering the same CV survey in two countries allowed us to explore the issue whether WTP to prevent CM deaths varies across different populations. We found that the respondents’ country did significantly predict WTP even after controlling for individual characteristics likely to be associated with WTP. The results from this study suggest that countries might be better served to design original CV studies to value the benefits of preventing CM if they want to conduct a BCA of prevention interventions.

The only way to parse out what really lies behind one population’s WTP to prevent CM death relative to another population’s WTP is to supplement these CV surveys with additional questions about how one defines CM and questions that begin to explain preferences and factors that influence WTP to prevent CM. At a minimum, the results from this study suggest that payment mechanism is one preference that may influence whether and how much one is willing to pay to prevent CM deaths and that this preference may hold across different cultures.

A real strength to this research, as with the study results reported from the comparison sample in the United States [14], is a benefits estimate for preventing a death associated with CM in Ecuador. That is, if each person is willing to pay more than $200 to reduce the number of deaths resulting from CM by 1 in 100,000 per year, then the value of one child’s life saved in a population of 100,000 is $20 million. This represents a significant value that society places on preventing a death from CM. Once this benefits estimate is validated nationally, and coupled with evidence of effective programs, policies and interventions to prevent CM mortality and their costs, these data will prove useful to policy makers considering how to allocate scarce public resources to improve the lives of children.
